# PLX8394, a new generation BRAF inhibitor, selectively inhibits BRAF in colonic adenocarcinoma cells and prevents paradoxical MAPK pathway activation

**DOI:** 10.1186/s12943-017-0684-x

**Published:** 2017-06-28

**Authors:** Candani S. A. Tutuka, Miles C. Andrews, John M. Mariadason, Paul Ioannidis, Christopher Hudson, Jonathan Cebon, Andreas Behren

**Affiliations:** 1 Olivia Newton-John Cancer Research Institute, 145 Studley Road, Heidelberg, VIC 3084 Australia; 2 Austin Medical Oncology Unit, Austin Health, Heidelberg, VIC Australia; 3 School of Cancer Medicine, La Trobe University, Bundoora, VIC Australia; 4 Department of Medicine, University of Melbourne, Parkville, VIC Australia

**Keywords:** BRAF, Melanoma, Colorectal cancer, Paradoxical activation, MAPK pathway

## Abstract

BRAF inhibitors (BRAFi) are standard of care for the treatment of *BRAF* V600 mutation-driven metastatic melanoma, but can lead to paradoxical activation of the mitogen-activated protein kinase (MAPK) signalling pathway. This can result in the promotion of precancerous lesions and secondary neoplasms, mainly (but not exclusively) associated with pre-existing mutations in *RAS* genes. We previously reported a patient with synchronous *BRAF*-mutated metastatic melanoma and *BRAF* ^*wt*^/*KRAS* ^G12D^-metastatic colorectal cancer (CRC), whose CRC relapsed and progressed when treated with the BRAF inhibitor dabrafenib (GSK2118436). We used tissue from the resected CRC metastasis to derive a cell line, LM-COL-1, which directly and reliably mimicked the clinical scenario including paradoxical activation of the MAPK signalling pathway resulting in increased cell proliferation upon dabrafenib treatment. Novel BRAF inhibitors (PLX8394 and PLX7904), dubbed as “paradox breakers”, were developed to inhibit V600 mutated oncogenic BRAF without causing paradoxical MAPK pathway activation. In this study we used our LM-COL-1 model alongside multiple other CRC cell lines with varying mutational backgrounds to demonstrate and confirm that the paradox breaker PLX8394 retains on-target inhibition of mutated BRAF V600 without paradoxically promoting MAPK signalling.

**Electronic supplementary material:**

The online version of this article (doi:10.1186/s12943-017-0684-x) contains supplementary material, which is available to authorized users.

## Introduction

A number of studies have demonstrated BRAFi-induced paradoxical activation, particularly when RAS is hyperactivated [1–4]. This is most commonly manifested as promotion of both benign and malignant hyperproliferative squamous cutaneous lesions in patients treated with BRAFi [5, 6]. Of greater concern is the increased incidence of secondary primary melanomas [7], and the reported emergence of RAS-driven cancers [8–10] including our own CRC case study in which combined treatment with dabrafenib and the MEK inhibitor trametinib (GSK 1120212, GlaxoSmithKline) was insufficient to block disease progression [11]. We subsequently established a cell line (LM-COL-1) from the colon cancer metastasis which was able to recapitulate what was observed in the patient during BRAFi treatment. The cell line provided us with a relevant model in which to investigate BRAFi and paradoxical MAPK activation.

In view of the frequency of *RAS* mutations in CRC [12] and pancreatic cancer [13], and the unknown prevalence of occult MAPK activating mutations in the population at large, it is anticipated that drug-promoted cancers will continue to emerge as a serious clinical problem in patients receiving BRAFi [1]. Consequently, a new generation of BRAFi termed “paradox breakers”, such as PLX8394 and PLX7904 (Plexxikon), has been developed [14–16].

## Findings

Firstly, we compared the on-target efficacy of PLX8394 (Plexxikon, Berkeley, CA) and the classical BRAFi, vemurafenib, by treating a *BRAF* ^***V600E***^ melanoma cell line, LM-MEL-64, and a *BRAF* ^*wt*^ */RAS* ^*wt*^ melanoma cell line, LM-MEL-39 with both drugs (Additional file 1: Material and Methods). Strong MAPK pathway inhibition in LM-MEL-64 was demonstrated by an 80.3 ± 2.4% (mean ± SD) reduction of pERK at the 1 μM dose relative to control, while little or no change in pERK was observed in LM-MEL-39 (Additional file 2: Figure S1).

**Fig. 1:**
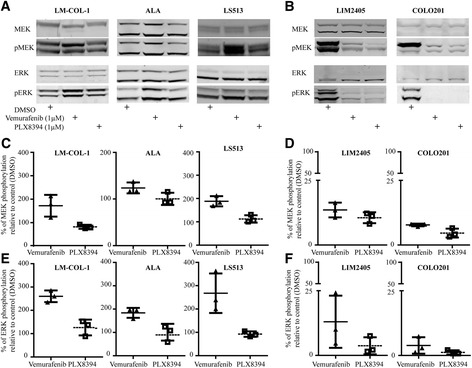
Effect of the BRAF inhibitors vemurafenib and PLX8394 on the MAPK pathway in colorectal cancer cell lines. Cells were treated with DMSO, vemurafenib at 1 μM, or PLX8394 at 1 μM for 6 h. **a**, **b** Representative Western blot of a panel of *BRAF* ^*wt*^ */KRAS* ^*G12D*^ (LM-COL-1, ALA, and LS513) and *BRAF* ^*V600E*^ */KRAS* ^*wt*^ (LIM2405 and COLO 201) colorectal cancer cell lines after treatment with DMSO control or BRAF inhibitors. Western blots were probed for total and phosphorylated MEK1/2 and ERK1/2. The blots are representative of three independent experiments. Total ERK served as a loading control. Western blot signal intensity was quantified and used to measure protein level relative to control. **c**, **d** Densitometry of MEK1/2 phosphorylation demonstrating paradoxical activation by vemurafenib in *KRAS*-mutated cell lines and BRAFi sensitivity in *BRAF* ^*V600E*^ mutated cell lines LIM2405 and COLO 201. **e**, **f** Densitometry of ERK1/2 phosphorylation in the same cell lines as shown in c and d. In panels **c**–**f** the total protein:phosphorylated ratio is expressed as the mean ± SD of three independent replicates relative to DMSO-treated control

Since paradoxical activation of MAPK signalling appeared to have driven the growth of the colorectal cancer in our CRC case study [11], we examined whether this could be replicated in the LM-COL-1 cell line and additional colorectal cancer cell lines with varying mutational status, and whether this effect could be mitigated by use of PLX8394. The cell lines and their mutational status used in this study are shown in Table 1. Consistent with our previous findings, the BRAFi vemurafenib induced a dose-dependent paradoxical increase in the levels of pMEK and pERK in LM-COL-1 at the 1 μM dose of 72.1 ± 24.5% and 160.2 ± 18.0% (mean ± SD), respectively. In contrast, treatment with the paradox breaker PLX8394 had minimal effect on pMEK and pERK in this cell line (Fig. 1a, c, and e). Similar effects could be seen in the two additional *BRAF* ^*wt*^ */ KRAS* ^*G12D*^ colon cancer cell lines, ALA and LS513 (Fig. 1a, c, and e), and were also observed when we applied the same treatments on the *BRAF* ^*wt*^ */ KRAS* ^***G13D***^ colon cancer cell line HCT 116 (Additional file 3: Figure S2). Conversely, both vemurafenib and PLX8394 decreased MEK1/2 and ERK1/2 phosphorylation in the *BRAF* ^*V600E*^ colon cancer cell lines LIM2405 and COLO 201 (Fig. 1b, d, and f).

**Table 1:** Mutational status of cell lines used

Cell Line	Type	BRAF	KRAS
LM-MEL-64	Melanoma	V600E	wt
LM-MEL-39	Melanoma	wt	wt
LM-COL-1	Colorectal carcinoma	wt	G12D
LS513 [21]	Colorectal carcinoma	wt	G12D
ALA [12]	Colorectal carcinoma	wt	G12D
LIM2405 [22]	Colorectal carcinoma	V600E	wt
COLO 201 [23]	Colorectal carcinoma	V600E	wt
HCT 116	Colorectal carcinoma	wt	G13D

*wt* wild type

**Fig. 2:**
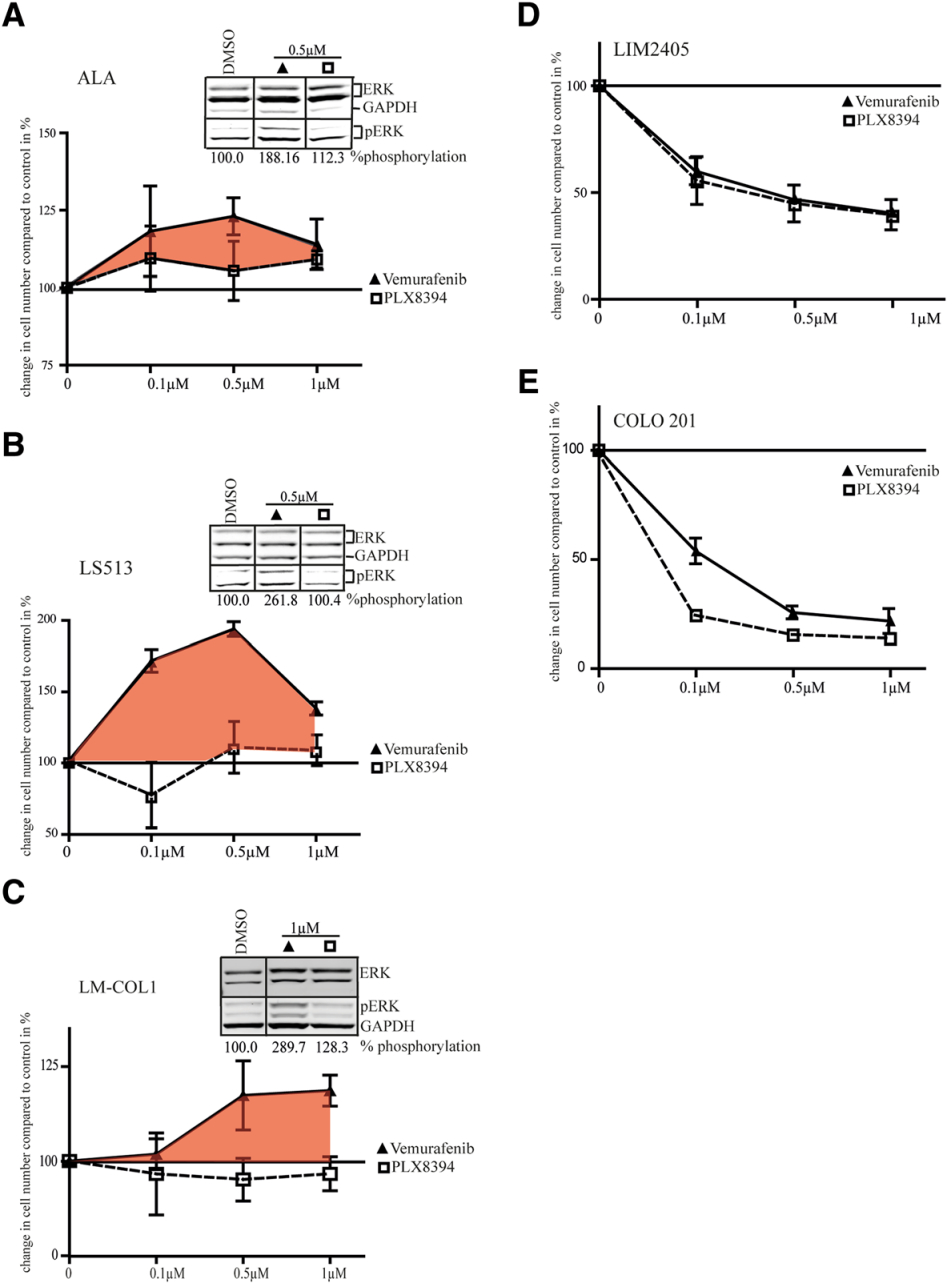
The effect of vemurafenib and PLX8394 on proliferation and survival of *BRAF* ^*wt*^ */ KRAS* ^*G12D*^ and *BRAF* ^*V600E*^ */ KRAS* ^*wt*^ colorectal cancer cell lines. Inhibitors were used at 0 (DMSO control), 0.1, 0.5, and 1 μM. Cell proliferation was measured after 72 h of BRAFi treatment. **a**–**c** Proliferation of *BRAF* ^*wt*^ */KRAS* ^*G12D*^ colorectal cancer cell lines after treatment with vemurafenib or PLX8394 at the indicated concentrations. Relative cell numbers are normalized to DMSO-treated control and differences shown as %. The tinted area indicates increased proliferation after treatment with vemurafenib. The Western blot inlay demonstrates the amount of ERK1/2 phosphorylation relative to the DMSO control at the concentration of vemurafenib that resulted in the biggest increase in proliferation. Lines between lanes denote non-adjacent lanes from the same membrane. **d**–**e** Inhibition of proliferation in *BRAF* ^*V600E*^ */ KRAS* ^*wt*^ colorectal cancer cell lines LIM2405 and COLO 201 after treatment with the indicated concentrations of vemurafenib or PLX8394. All data are shown as mean ± SD of independent triplicates relative to DMSO-treated controls

To assess the functional effects of these inhibitors, proliferation assays were performed after 72 h treatment with either vemurafenib or PLX8394 across a range of concentrations. Consistent with the increase in MAPK signalling, proliferation of ALA, LS513, LM-COL-1, and HCT 116 was enhanced when treated with vemurafenib, but not with PLX8394 (Fig. 2a–c, and Additional file 3: Figure S2d). Notably, the largest effect on vemurafenib-induced cell proliferation was observed at the clinically achievable dose of 0.5 μM for ALA and LS513. Western blot inlays from signalling analysis of vemurafenib at concentrations that resulted in the greatest effect of increased proliferation, 0.5 μM for ALA and LS513, 1 μM for LM-COL-1, and 0.1 μM for HCT 116, demonstrate paradoxical increase of pERK in these cell lines (Fig. 2a–c, and Additional file 3: Figure S2a, b and c).

Conversely, *BRAF* ^*V600E*^ colon cancer cell lines LIM2405 and COLO 201 showed a decrease in pMEK and pERK levels with treatment (Fig. 1b, d, and f), consistent with reduced proliferation with both inhibitors (Fig. 2d and e).

## Conclusions

We demonstrate that paradoxical activation of MAPK signalling and the consequent promotion of proliferation of *KRAS*-mutated colon cancer cells is markedly reduced by newer-generation paradox-breaker BRAF inhibitors, while their capacity to inhibit mutant BRAF-driven signalling is not compromised. Mechanistically, it has been demonstrated that this may be a consequence of the minor structural changes between paradox breakers and vemurafenib which most likely avoid paradoxical activation of MAPK signalling by preventing RAF dimer formation [16].

Amaravadi et al. reported that long-term BRAFi treatment can enhance neoplastic growth in colonic cells harbouring mutations of the tumour suppressor gene, adenomatous polyposis coli (*APC*), even without *KRAS* mutations [17]. While patient numbers in this study were small, 4 out of the 14 patients treated with conventional BRAFi presented with 5 or more colonic polyps, significantly increasing the potential risk for progression to colon cancer [18, 19]. Furthermore, using the *APC Min +/−* model, the authors demonstrated an increased number and shorter time to appearance of polyps in mice treated with vemurafenib compared with control. Collectively, these data emphasize the risks associated with long-term BRAFi treatment, and the applicability of these risks even to those patients with no prior history of *RAS* mutated cancer.

To sustain inhibition of MAPK signalling and to overcome paradoxical MAPK activation, BRAF inhibitors have been tested in combination with MEK inhibitors. Whilst this combination has been shown to improve survival times of patients with *BRAF* mutated melanoma [20], it is noteworthy that two of the cases of occult *RAS* mutated tumour progression on BRAFi therapy received, at least at some time-point, the BRAFi/MEKi combination [8, 11]. This suggests that the addition of a MEK inhibitor cannot completely abrogate BRAFi induced paradoxical MAPK activation. The mechanism of the paradox breaker’s ability to avoid paradoxical activation of the MAPK pathway has been previously demonstrated by Zhang et al. [16], using multiple cell lines including HCT 116. While the study showed similar paradoxical activation with vemurafenib in this cell line as demonstrated here, we extended these findings to additional colorectal carcinoma cell lines with different mutational backgrounds. Moreover, our study shows functional consequences of the paradoxical activation for the growth rate of the colorectal cancer cell lines, with a slight but consistent increase detected with vemurafenib treatment.

Overall, our findings justify the evaluation of paradox breaker BRAF inhibitors as the next generation of therapeutics for the treatment of *BRAF* mutated cancers. It suggests that the new paradox breakers have the potential to mitigate the risk of promoting occult *RAS* activated tumour progression associated with the use of the first generation BRAFi, without compromising therapeutic efficacy or narrowing the therapeutic window. Currently, PLX8394 is undergoing a clinical trial in solid unresectable tumors (NCT02428712) with a completion date of 2018, at which time point the usefulness of these drugs in the clinical setting will become clear.

## Additional files

Additional file 1: Material and Methods. (DOCX 19 kb)

Additional file 2: Figure S1. The effect of vemurafenib and PLX8394 on the *BRAF* ^*V600E*^ melanoma cell line LM-MEL-64 and the *BRAF* ^*wt*^ melanoma cell line LM-MEL-39. (A) LM-MEL-64 and (B) LM-MEL-39 were treated with the indicated concentrations of vemurafenib or PLX8394 and immunoblotting for total and phosphorylated ERK was performed. (C) pERK densitometry relative to control expressed as (%) ± SD for LM-MEL-64 and (D) for LM-MEL-39. Data are from three independent experiments. (TIFF 342 kb)

Additional file 3: Figure S2. The effect of BRAF inhibitors vemurafenib and PLX8394 on *BRAF* ^*wt*^ */ KRAS* ^*G13D*^ cell line HCT 116**.** Cells were treated with DMSO, vemurafenib at 1 μM, or PLX8394 at 1 μM for 6 h. (A) Representative Western blot after treatment with DMSO control or BRAF inhibitors. Western blots were probed for total and phosphorylated MEK1/2 and ERK1/2. The blots are representative of three independent experiments. GAPDH served as a loading control. Western blot signal intensity was quantified and used to measure protein level relative to control. (B) Densitometry of MEK1/2 phosphorylation demonstrating paradoxical activation by vemurafenib in HCT 116. (C) Densitometry of ERK1/2 phosphorylation in the same cell line. Total protein:phosphorylated protein ratio is expressed as the mean ± SD of three independent replicates relative to DMSO-treated control. (D) Inhibitors were used at 0 (DMSO control), 0.1, 0.5, and 1 μM. Cell proliferation was measured after 72 h of BRAFi treatment. Relative cell numbers are normalized to DMSO-treated control and differences shown as percentage. The tinted area indicates increased proliferation after treatment with vemurafenib. The Western blot inlay demonstrates the difference in ERK1/2 phosphorylation at the concentration of vemurafenib that resulted in the biggest increase in proliferation. (TIFF 1052 kb)
