# Supplementary material for: PLX8394, a new generation BRAF inhibitor, selectively inhibits BRAF in colonic adenocarcinoma cells and prevents paradoxical MAPK pathway activation
Source: Mol Cancer. 2017 Jun 28;16:112. doi: 10.1186/s12943-017-0684-x (PMC5490236; doi:10.1186/s12943-017-0684-x)
Supplement: Supplementary file 1 — Material and Methods. (DOCX 19 kb) [file 12943_2017_684_MOESM1_ESM.docx]

**Tutuka *et al.*** **PLX8394, a new generation BRAF inhibitor, selectively inhibits BRAF in colonic adenocarcinoma cells and prevents paradoxical MAPK pathway activation**

**additional File 1**

**Material and Methods**

**Cell lines**

The melanoma cell lines LM-Mel-64, LM-Mel-39, and the colorectal cancer cell line LM-COL-1 were derived in-house from patient-derived melanoma metastases (LM-Mel-64 and -39) and a colon cancer metastasis (LM-COL-1) as previously described. All cell lines were mycoplasma-tested in-house and found to be negative. All melanoma cell lines and LM-COL-1 were matched to their respective donors by HLA-typing (Red Cross, Melbourne, VIC, Australia). All cell lines were authenticated by STR profiling.

In the CRC panel, the ***BRAF*** wild type, ***KRAS*** mutant cell lines ALA and LS513 were provided by Diego Arango (CIBBIM – Nanomedicine, Vall d’Hebron University Hospital Research Institute, Barcelona, Spain) and by Oliver Sieber (Walter and Eliza Hall Institute, Melbourne, Australia) respectively. The ***BRAF*** mutant and ***KRAS*** wild type cell line COLO 201 and the ***BRAF*** wild type and ***KRAS*** mutant cell line HCT 116 was purchased from ATCC (Manassas, VA, USA) and LIM2405 was provided by the Ludwig Institute for Cancer Research, Parkville branch, (Melbourne, Australia).

**Cell culture**

All tissue culture media was supplemented with 1% glutamine (Glutamax, Life Technologies, Australia), 1% penicillin/streptomycin antibiotic mix (Penstrep, Life Technologies ^TM^) and heat-inactivated fetal bovine serum (FBS; Sigma). LM-COL-1 was grown in RPMI-1640 media (Life Technologies™) supplemented with 15% FBS. LM-Mel-64 and LM-Mel-39 were grown in RPMI with 10% FBS. LS513, LIM2405, HCT 116 were grown in DMEM containing 10% FBS, whilst ALA and COLO 201 were grown in DMEM-F12 containing 10% FBS. All culture media was sterile-filtered. Cells were incubated at 37°C in 5% CO_2_ atmosphere (MCO-18AIC, SANYO Electric Biomedical Co. Ltd., Japan). At early passage, cell lines were split into three separate tissue culture flasks and subsequently passaged independently serving as three independent replicates.

**Chemicals**

PLX4032 and PLX8394 were generously provided by Dr Gideon Bollag (Plexxikon, Berkeley CA, USA). Concentrated stock solutions of inhibitors were prepared in dimethyl sulfoxide (DMSO, Sigma).

**Cell treatment for signalling analysis**

Cells were initially serum-starved overnight in 2% FBS except for LS513, which was incubated overnight in FBS-free media containing sterile-filtered 0.5% bovine serum albumin (Sigma, Cat No 05470). Cells were treated *in vitro* with fresh medium containing inhibitors at 0.1, 0.5 and 1 µM or DMSO-only control (0.1%) for 6 hours prior to harvest for Western blot analysis.

**Protein Extraction and Quantitation**

Cells were lysed in RIPA buffer (Thermo Fisher Scientific Inc., USA) with added protease and phosphatase inhibitors (cOmplete^TM^ ULTRA and PhosSTOP^TM^, Roche Applied Bioscience, Germany). Total protein content of cell lysates was determined using the Pierce® BCA Protein Assay kit (Thermo Fisher Scientific Inc., USA) following the manufacturer’s protocol.

**Western Blot and Densitometry**

Protein separation was performed using NuPAGE® Novex® Bis-Tris 4-12% pre-cast electrophoresis gels in NuPAGE® MOPS SDS Running Buffer, and sized using the SeeBlue® Plus2 pre-stained protein standard (Novex®, Life Technologies^TM^, Carlsbad CA, USA). Protein gels were transferred onto nitrocellulose membranes using the iBlot protein transfer system (Invitrogen^TM^). Western blots were probed using antibodies against total ERK1/2 (mouse anti-p44/42 MAP-Kinase (Erk1/2) (3A7) mAb #9107), phospho-ERK1/2 (rabbit anti-phospho-p44/42 MAP-Kinase (Erk1/2)(Thr202/ Tyr204) mAb #4377), total MEK1/2 (mouse anti-Mek1/2 (L38C12) mAb #4694), phospho-MEK1/2 (rabbit anti-phospho-Mek1/2 (Ser217/221)(41G9) mAb #9154), GAPDH (rabbit anti-GAPDH (14C10) mAb#2118), and GAPDH (mouse anti-GAPDH (D4C6R) mAb #97166) all from Cell Signaling Technologies. Target proteins were then visualised with secondary conjugated antibodies IRDye® 680RD goat anti-mouse IgG and IRDye® 800CW goat anti-rabbit IgG (LI-COR®, Prod. Nos. 926-68070 and 926-32211). Antibody solutions were prepared at 1:1000 for primary and 1:10,000 for secondary antibodies following the manufacturer’s recommendation. Visualization and analysis of target protein was performed with the LI-COR® ODYSSEY® Infra-Red Scanning System (LI-COR®). Signal intensity was analysed using the analytical feature of the LI-COR ODYSSEY software keeping signal area and measurement method consistent. For phospho-protein analysis, the ratio of phosphorylated to total protein was determined and normalised to the untreated (DMSO) control for each cell line to compare relative changes in phosphorylation status of target proteins.

**Cellular proliferation assay**

Cellular proliferation assay of colon cancer cell lines was performed on Falcon^®^ 96 well clear plates. Cells were seeded at 3000 cells per well. Cells were incubated with the inhibitors vemurafenib or PLX8394 at concentrations of 0 µM (DMSO), 0.1 µM, 0.5 µM, and 1 µM. Proliferation assays were performed using the MTS-based CellTiter 96® AQueous One Solution Cell Proliferation Assay (Promega®, Madison WI, USA). Briefly, cells were incubated for 1 hour in diluted CellTiter mixture (1:5 dilution in growth media) prior to measurement of absorbance at 490nm wavelength (VERSAmax, Molecular Devices, Sunnyvale CA, USA) and subtraction of background as measured from no-cell control wells. Readings were performed at day 1 before treatment and after 72 hours (3 days) of inhibitor treatment. Absorbance were normalised within cell lines to untreated controls.

**Statistical analysis**

Statistical analysis of signal densitometry was done using a Two-tailed *t* test as comparisons between two experimental groups. *P* value off <0.05 is considered significant. Bars show mean ±SD. Analysis was performed using GraphPad Prism Software.
