# Supplementary figures and images for: PLX8394, a new generation BRAF inhibitor, selectively inhibits BRAF in colonic adenocarcinoma cells and prevents paradoxical MAPK pathway activation
Source: Mol Cancer. 2017 Jun 28;16:112. doi: 10.1186/s12943-017-0684-x (PMC5490236; doi:10.1186/s12943-017-0684-x)

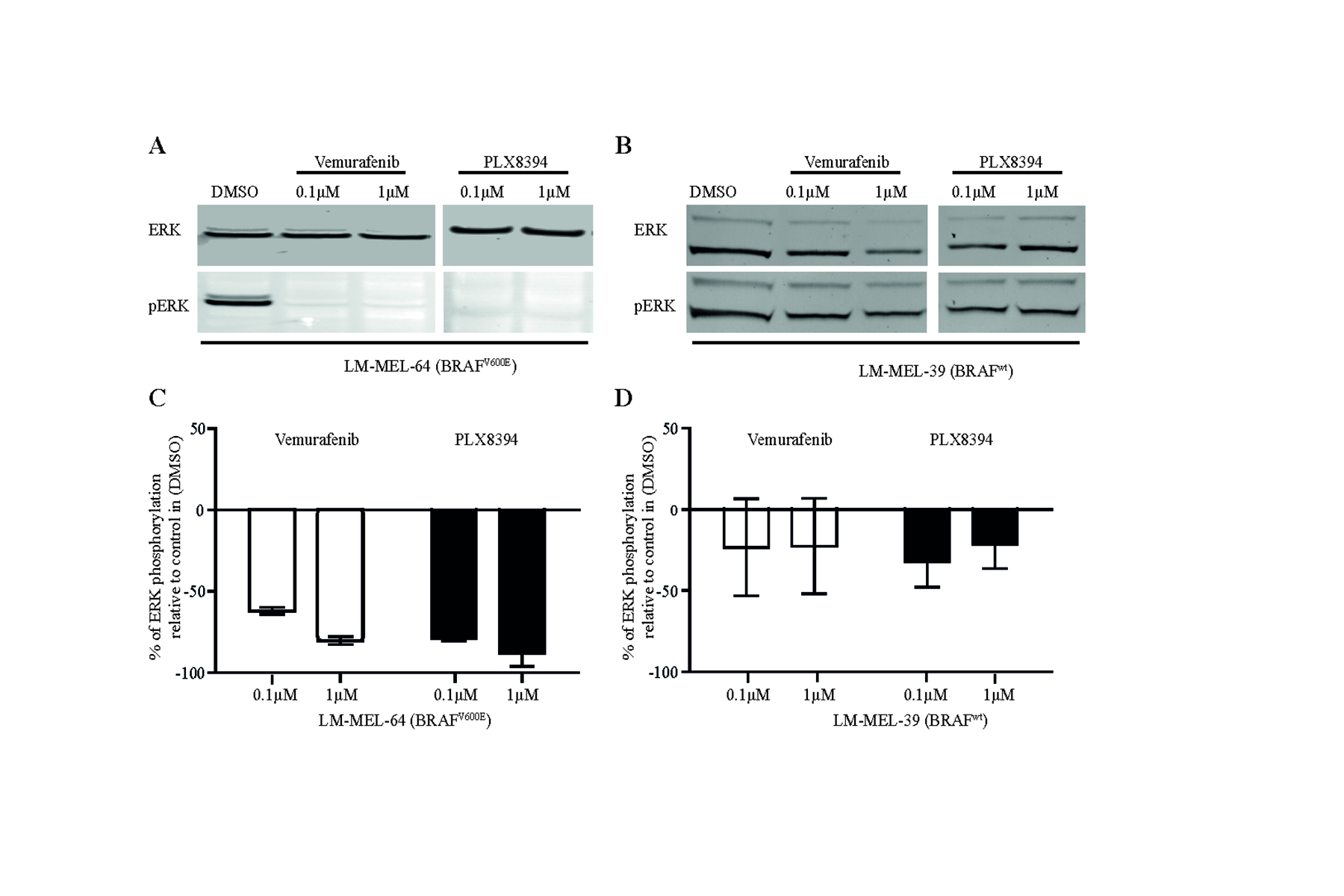

Supplement: Supplementary file 2 — The effect of vemurafenib and PLX8394 on the BRAF V600E melanoma cell line LM-MEL-64 and the BRAF wt melanoma cell line LM-MEL-39. (A) LM-MEL-64 and (B) LM-MEL-39 were treated with the indicated concentrations of vemurafenib or PLX8394 and immunoblotting for total and phosphorylated ERK was performed. (C) pERK densitometry relative to control expressed as (%) ± SD for LM-MEL-64 and (D) for LM-MEL-39. Data are from three independent experiments. (TIFF 342 kb) [file 12943_2017_684_MOESM2_ESM.tif]

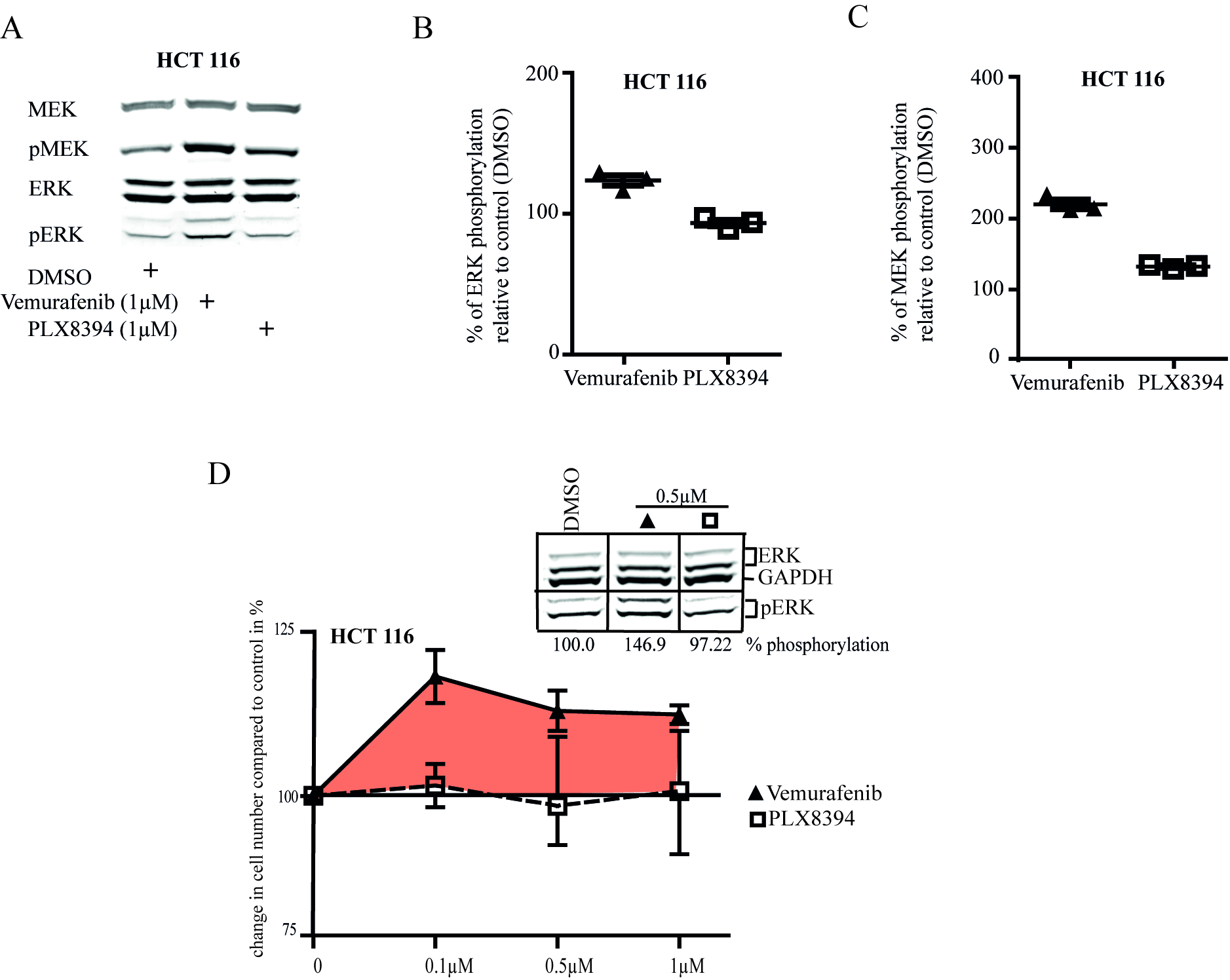

Supplement: Supplementary file 3 — The effect of BRAF inhibitors vemurafenib and PLX8394 on BRAF wt / KRAS G13D cell line HCT 116. Cells were treated with DMSO, vemurafenib at 1 μM, or PLX8394 at 1 μM for 6 h. (A) Representative Western blot after treatment with DMSO control or BRAF inhibitors. Western blots were probed for total and phosphorylated MEK1/2 and ERK1/2. The blots are representative of three independent experiments. GAPDH served as a loading control. Western blot signal intensity was quantified and used to measure protein level relative to control. (B) Densitometry of MEK1/2 phosphorylation demonstrating paradoxical activation by vemurafenib in HCT 116. (C) Densitometry of ERK1/2 phosphorylation in the same cell line. Total protein:phosphorylated protein ratio is expressed as the mean ± SD of three independent replicates relative to DMSO-treated control. (D) Inhibitors were used at 0 (DMSO control), 0.1, 0.5, and 1 μM. Cell proliferation was measured after 72 h of BRAFi treatment. Relative cell numbers are normalized to DMSO-treated control and differences shown as percentage. The tinted area indicates increased proliferation after treatment with vemurafenib. The Western blot inlay demonstrates the difference in ERK1/2 phosphorylation at the concentration of vemurafenib that resulted in the biggest increase in proliferation. (TIFF 1052 kb) [file 12943_2017_684_MOESM3_ESM.tif]
